# Automated total nucleic acid extraction with magnetic beads for the detection of *Plasmodium falciparum* in large study cohorts

**DOI:** 10.1186/s12936-024-05227-z

**Published:** 2024-12-23

**Authors:** Juliana Inoue, Nina Huber, Rolf Fendel, Jana Held

**Affiliations:** 1 Institute of Tropical Medicine, Eberhard Karls University of Tübingen, Tübingen, Germany; 2 German Center for Infection Research (DZIF), Partner Site Tübingen, Tübingen, Germany; 3 Centre de Recherches Médicales de Lambaréné (CERMEL), Lambaréné, Gabon

**Keywords:** *Plasmodium*, Nucleic acid extraction, qPCR, Automation

## Abstract

**Background:**

Molecular methods play an important role in clinical trials assessing anti-malarial drugs and vaccines, as well as in epidemiological studies aimed at detecting *Plasmodium* species, especially when dealing with large sample sizes. Molecular techniques are more sensitive and generally have a higher throughput compared to the gold standard microscopy. Further optimization can be achieved with automation of nucleic acid isolation, allowing for rapid and precise extraction. This study evaluated the isolation of total nucleic acids from *Plasmodium falciparum* mocked samples using an automated extraction method with a magnetic bead-based kit compared to a manual silica column-based kit. Additionally, two different RNA preservation solutions were compared.

**Methods:**

*Plasmodium falciparum* Dd2 parasites were serially diluted and spiked into whole blood. The dilutions were stored in two different RNA preservation solutions and total nucleic acids extracted with an automated magnetic bead-based kit and a manual silica column-based kit. Subsequently, a reverse transcription (RT) qPCR for *Plasmodium* detection targeting *Plasmodium* 18S rRNA and DNA in a single reaction was performed and the quantification cycle (Cq) values across the different sample groups were compared.

**Results:**

Comparable Cq values across the various sample preparations were obtained, suggesting minimal influence from RNA preservation solutions (p = 0.686) or extraction methods (p = 0.119) on RT-qPCR outcomes. Automated nucleic acids extraction allowed processing numerous samples in a shorter timeframe and showed similar efficiency in detecting *Plasmodium* in blood samples by RT-qPCR as manual extraction.

**Conclusions:**

The automated method for nucleic acid isolation is a valuable tool for the detection of *Plasmodium* infections in large-scale studies. It is efficient, reliable, and cost-effective. Its potential applications extend to other molecular surveillance studies to support malaria control measures.

## Background

Malaria remains a public health concern with estimated 249 million cases and 608,000 deaths worldwide in 2022 [1]. Sub-Saharan Africa is the most affected region, accounting for 95% of cases and deaths, mainly in children under five. The analysis of thick blood smear by microscopy remains the gold standard method for malaria diagnosis. It is a relatively low-cost technique but requires well trained personnel [2]. A microscopist from a reference laboratory is able to detect five parasites (p)/µL, however, in the routine 50 p/µL are usually detected [2]. Rapid diagnostic tests (RDTs) represent an alternative diagnostic method as they require less training and are easy to perform [3]. However, the RDTs available on the market usually only detect a parasitaemia above 200 p/μL [4], do not quantify parasitaemia, and present a low specificity and sensitivity for non-falciparum species. Additionally, certain *Plasmodium falciparum* strains can evade RDT detection by not expressing the histidine-rich proteins 2 and 3 (HRP2/3), the most commonly targeted antigens in RDTs [5]. Both methods (microscopy and RDTs) are suitable for individual diagnosis, but for epidemiological and clinical studies with large sample sizes molecular methods are more appropriate, especially when investigating e.g. multiplicity of infection [6, 7], population structure [8], drug and diagnostic resistance [9, 10], where identification of minor clones is critical, or in case of low-level or sub-microscopic parasitaemia.

The use of methods with increased specificity and sensitivity is of especial importance in the context of malaria elimination and eradication, where the identification of possible reservoirs becomes crucial, often involving individuals with sub-microscopic and/or asymptomatic infections. The use of polymerase chain reaction (PCR) for malaria diagnosis was first reported in 1990 [11], and a decade later quantitative real-time PCR (qPCR) was described [12], markedly improving the limit of detection for *Plasmodium* infections. *Plasmodium* genes with multiple copies like the 18S ribosomal RNA (rRNA) [13], *cytB* [14] in the mitochondrial genome, telomere-associated repetitive element 2 (TARE-2) and the *var* gene acidic terminal sequence (*var*ATS) [15] are usually targeted for PCR to increase sensitivity. For the 18S rRNA gene, reverse transcriptase (RT)-qPCR can enhance the limit of detection, as a single asexual parasite has thousands of 18S rRNA transcripts [16]. In addition, process automation can further improve sample throughput in large-scale studies when applied to nucleic acids extraction as well as downstream applications, such as PCR. A widely used method for blood sampling in large-scale studies is dried blood spots (DBS), consisting of drops of blood on filter paper that can be stored and transported at room temperature and require minimal storage volume. Nevertheless, DNA recovery rates from DBS can be 5- to 10-times lower than from whole blood samples [17].

In this study, an automated nucleic acids extraction from whole blood was evaluated as a high-throughput method for detection of *P. falciparum* by 18S RT-qPCR. To achieve this, manual extraction by a silica membrane column-based kit was compared with an automated magnetic-based nucleic acid extraction kit. In addition, two different RNA preservation solutions were evaluated. To assess the nucleic acid yield from whole blood samples for *P. falciparum* detection, the quantification cycle (Cq) values obtained by RT-qPCR across the different sample groups were compared.

## Methods

This study compared a silica membrane column-based manual kit and an automated magnetic bead-based kit for their ability to recover nucleic acids from whole blood and further use for *Plasmodium* spp. detection by a reverse transcription quantitative PCR. In addition, two RNA preservation solutions were compared: RNAlater® (Thermo Fisher Scientific) and DNA/RNA Shield™ (Zymo Research).

### Nucleic acid extraction systems

The QIAamp DNA Blood Mini Kit (Qiagen) is a commercial kit using guanidine-based lysis buffer and a silica membrane with affinity to nucleic acids. After a lysis step of 10-min incubation at 56 °C, nucleic acids are precipitated with ethanol. Next, samples are loaded into the silica membrane column and centrifuged, allowing the membrane to retain the nucleic acids. Two steps of washing are carried out by centrifugation at room temperature. Elution is also performed by centrifugation at room temperature.

The sbeadex blood kit (LGC) is a magnetic bead-based kit containing double coated superparamagnetic beads to which the nucleic acids bind in a two-step binding mechanism. In this study, the sbeadex kit was automated using the KingFisher Flex System (Thermo Fisher Scientific) to shorten the time needed for extraction. This machine allows for the automation of binding, washing and elution of the magnetic beads, and at the same time minimizes the risk of contamination since few user interventions are required during the process. The system consists of a magnetic head and a circular plate with slots for 96-well plates that rotates at each step of the extraction. The lysis step is performed at 60 °C for 20 min with constant shaking. Next, the magnetic beads are added to the lysed samples and the binding step is carried out at room temperature with constant shaking for 5 min. Beads and the nucleic acids complex are washed in three steps. The final elution step is carried out with incubation at 60 °C for 10 min and shaking. In a single run, 96 samples can be processed.

### Sample preparation

*Plasmodium falciparum* Dd2 parasites were maintained in culture in complete culture medium, at 2.5% haematocrit in an incubator at 37 °C and 90% N2, 5% O2, 5% CO2 atmosphere. To be used in the experiment, parasites were synchronized to ring stage using magnetic MACS^®^ columns (Milteny Biotec) and the parasitaemia counted by microscopy. Next, the parasitaemia was diluted with medium to 125,000 p/µL and a five-fold serial dilution was performed with RPMI medium down to 0.064 p/µL, resulting in a total of 10 dilutions. 130 µL of each dilution was spiked into 1170 µL human whole blood from a naïve malaria person collected in an EDTA tube. This resulted in 10 different samples with a final parasitaemia ranging from 12,500 p/µL to 0.0064 p/µL. Each sample was divided and then stored in one of the two different RNA preservation solutions: RNAlater (250 µL infected whole blood plus 650 µL RNAlater) or DNA/RNA Shield 2x (250 µL blood plus 250 µL DNA/RNA Shield 2x). As a last step, each preparation was divided into two tubes with equal volumes and kept at − 20 °C for at least three weeks until performance of the nucleic acid purification. Non-infected whole blood was used as negative control in each sample preparation and purification method.

### DNA/RNA purification

#### Samples in RNAlater

The samples were thawed at room temperature and mixed by pipetting up and down. Of each sample 360 µL were pipetted in a new tube and centrifuged for 3 min at maximum speed before the supernatant was removed and discarded. PBS 1 × was added to 200 µL total volume for purification with QIAamp and sbeadex + KingFisher. The volumes of all reagents were used according to the manufacturer´s instructions. Elution was performed in 100 µL with both kits and nucleic acids were stored at – 20 °C until use.

#### Samples in DNA/RNA Shield

The samples were thawed at room temperature and mixed by pipetting up and down. Since samples stored in DNA/RNA Shield can be directly extracted without removal of the preservation reagent, 200 µL of the mixture was directly used for extraction in both kits. In this way, the blood volume used for nucleic acids extraction for samples preserved in RNAlater and DNA/RNA Shield was 100 µL and, therefore, the same in both methods. The volumes of all reagents were used according to the manufacturer´s instructions. The final elution was performed in 100 µL with both kits and nucleic acids were stored at − 20 °C until use. Nucleic acids extraction was confirmed by measuring RNA and DNA concentrations using the NanoDrop^®^ Spectrophotometer (Thermo Fisher Scientific).

### Reverse transcription quantitative polymerase chain reaction

RT-qPCR was performed for *Plasmodium* detection targeting *Plasmodium* 18S RNA and DNA in a single reaction. Reactions were carried out with TaqMan^®^ RNA-to-Ct™ 1-Step Kit (Thermo Fisher Scientific) and primers and probe previously published [18] with a minor modification in the probe: 5′ HEX fluorophore and 3′ Eclipse Quencher attached to the minor groove binder molecule. The RT-qPCR method used here was chosen based on its higher sensitivity compared to DNA-based assays. It reported a limit of detection of 0.002 p/µL [19]. Final volume reaction was 10 μL, with 2.5 μL total nucleic acids, 400 nM each primer and 150 nM probe. Cycling conditions were: 48 °C for 20 min, 96 °C for 10 min, and 45 cycles of 95 °C for 15 s, 62 °C for 1 min. Samples were assayed in triplicates along with no-template, positive and no-reverse transcriptase controls on the LightCycler 480 Instrument II (Roche Applied Science). The Cq value, i.e., the quantification cycle number that intersects the threshold line, was calculated using the second derivative maximum method (LightCycler 480 software; version 1.5.1.62) and the mean Cq of triplicates was calculated. The qPCR efficiency was determined from the slope of the standard curve from a tenfold serial dilution of *P. falciparum* total nucleic acid, ranging from 600,000 p/µL to 6 p/µL, with the following formula: E = −1 + 10^(−1/slope)^, where E is the qPCR efficiency.

### Statistical analysis

The statistical analyses were computed with R (version 4.2.2). A linear regression was performed to calculate the efficiency of the RT-qPCR. The Shapiro–Wilk test was done to test for normality and a three-way ANOVA to check the effect of the preservation solutions and extraction methods on the mean Cq values. Statistical significance was accepted when p-value ≤ 0.05.

### Calculation of the time required for sample processing

To estimate the time in minutes needed for sample processing by the automated method, the following formula was generated:

$$t\left( n \right) = 60\left( {\left\lfloor \frac{n}{96} \right\rfloor + w} \right) + 5{ }\left( {\left\lfloor \frac{n}{8} \right\rfloor + r} \right) + n*0.5 ,{ }w = \left\{ {\begin{array}{*{20}c} {0, if \,n\,mod\,96 = 0} \\ {1, if\,n\,mod\,96 > 0} \\ \end{array} { },{ }} \right.$$

$$r= \left\{\begin{array}{c}0, if\,n\,mod\,8=0\\ 1, if\,n\,mod\,8>0 \end{array}\right. n\in N$$

where $\left\lfloor {\frac{x}{{\text{y}}}} \right\rfloor$ denotes an integer division, also called floor function, in which the fractional part is discarded in the division [20]; 60 is the time in minutes that the robot takes to perform the extraction, 96 is the maximum number of samples that can be assessed in one round of extraction, 5 is the time in minutes needed for preparation of plates with reagents prior to robot initiation, 8 is the maximum number of samples that can be prepared using a multichannel pipette for the preparation of plates, and 0.5 is the time in minutes needed for pipetting each sample in the plate.

The following formula was generated to estimate the time required for the manual extraction method:

$$t\left(n\right)=17+7*n\,\,\,n\in N$$

In this formula, 17 is the time in minutes needed for the extraction steps that are not altered by the number of samples (as lysis incubation and centrifugation times), and 7 is the time in minutes that can vary depending on the number of samples.

## Results

As described in the method section, ten samples from the same *P. falciparum* serial dilution were evaluated in each sample preparation, totalizing 40 paired samples. The parasitaemia ranged from 12,500 to 0.0064 p/µL. For comparison of Cq values, samples were grouped according to the RNA preservation solution and extraction method used. Therefore, the following four groups were analysed: (1) samples in RNA later extracted automatically with magnetic beads (later/automated); (2) samples in RNAlater extracted manually with a silica column kit (later/manual); (3) samples in DNA/RNA Shield extracted automatically with magnetic beads (shield/automated); and (4) samples in DNA/RNA Shield extracted manually with a silica column kit (shield/manual).

### qPCR efficiency

The efficiency of the qPCR was 92% when calculated with a serial dilution of *P. falciparum* DNA ranging from 600,000 p/µL to 6 p/µL.

### Cq value comparison of positive samples from the four sample preparation groups

**Fig. 1:**
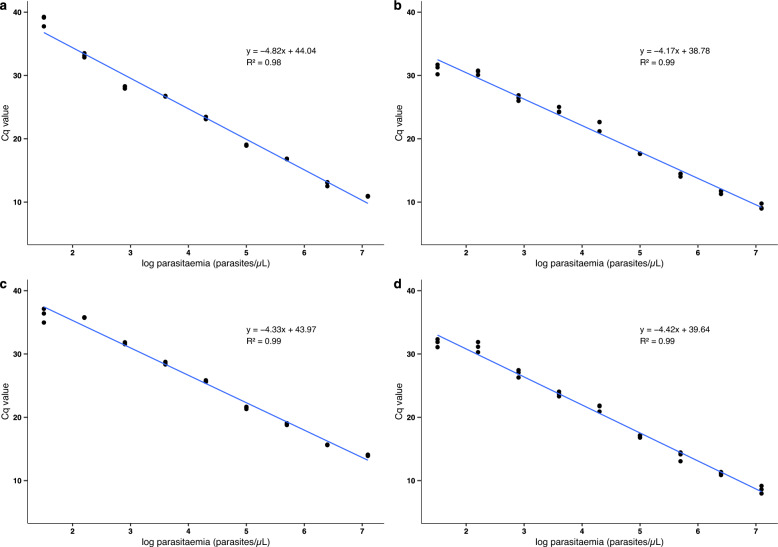
Quantification cycle (Cq) values from *P. falciparum* 18S RT-qPCR of 4 different sample preparation methods: **a** *P. falciparum* blood preserved in RNAlater extracted manually; **b** in RNA later extracted automatically; **c** in RNA/DNA Shield extracted manually and **d** in RNA/DNA Shield extracted automatically. Manual or automated nucleic acids isolation and RNA preservation solution have no effect on the mean Cq values obtained from RT-qPCR targeting 18S rRNA and DNA of *Plasmodium* (three-way ANOVA, p = 0.119, p = 0.686, respectively)

Following nucleic acid isolation, the samples were subjected to 18S Pan-*Plasmodium* RT-qPCR analysis. The limit of detection in all preparations was 0.032 p/µL. The Cq values obtained in the qPCR for each sample group are presented in Fig. 1. The influence of the preservation solution and the extraction method on the Cq values across different dilutions was evaluated by a three-way ANOVA. No significant effect was observed for either the preservation solution (p = 0.686) or the extraction method (p = 0.119). However, mean Cq values were higher when extracting nucleic acids with the column kit.

### Time and cost estimates for sample processing using automated and manual extraction methods

**Fig. 2:**
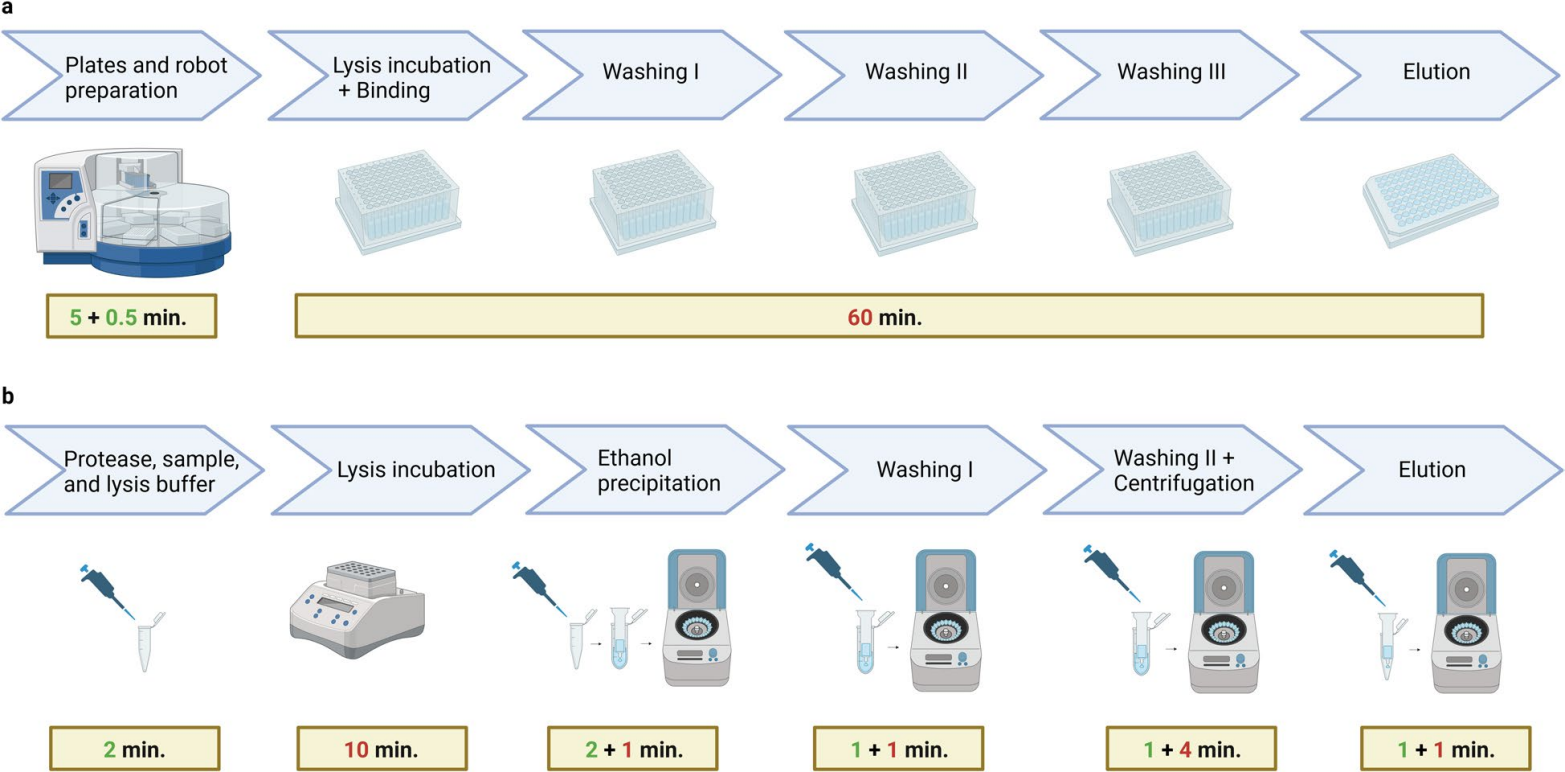
Comparison of the workflow for nucleic acid extraction of one sample using (**a**) an automated magnetic bead-based kit and (**b**) a manual silica column-based kit. The numbers inside the rectangles indicate the time in minutes (min.) for steps that take longer as the number of sample increases (in green) and for fixed times that are not influenced by the number of samples assessed (in red). Figure created with BioRender

**Fig. 3:**
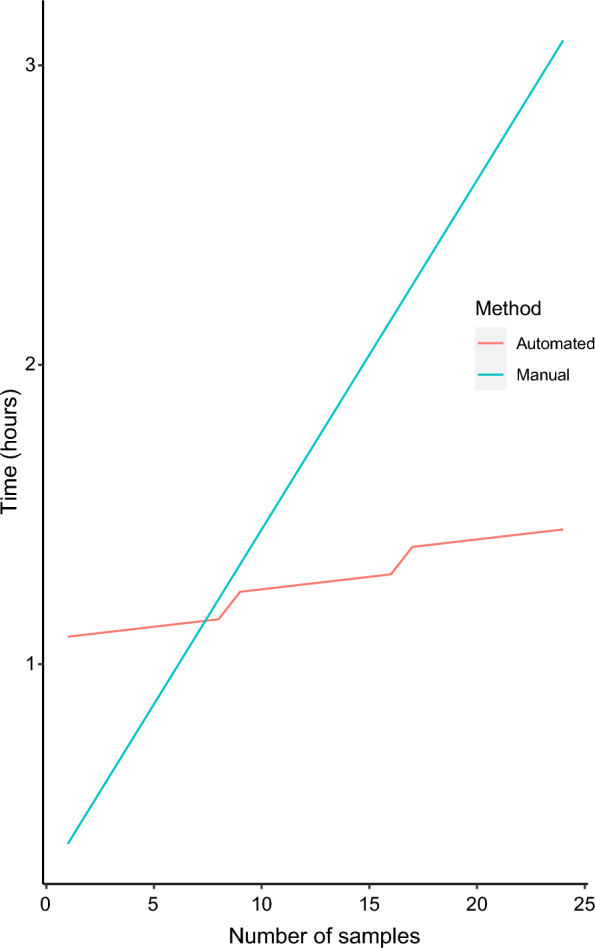
Estimated time for sample processing using two different methods for isolation of total nucleic acids: automated magnetic bead-based kit (red line) and manual silica column-based kit (blue line). The maximum number of samples that can be processed using a multichannel pipette for plate preparation is 8, therefore every 8 samples a step in the red line occurs representing the additional time needed. The automated method becomes more time-efficient than the manual method when at least 8 samples are processed at a time

The isolation of total nucleic acids using the automated method took around 60 min once the robot was loaded with samples and reagents, for up to 96 samples. With an increase in the number of samples, the time needed for preparation increased in approximately 5 min increments for every 8 samples, until reaching 96 samples, the maximum capacity of the robot (Fig. 2a). In contrast, extraction with the manual kit involves many steps that become longer as the number of samples increases (Fig. 2b). Processing a small number of samples took less time with the manual kit compared to the automated method. However, the processing time with the manual kit increased linearly with the number of samples. Consequently, the automated method became more time-efficient when processing at least 8 samples together (Fig. 3).

The costs for processing 96 samples considering the purchase of the extraction kits and specific plastic consumables were estimated. Extraction with the column-based kit costs 1.7 time as much as with the automated method (~ 368€ vs ~ 211€ for 96 samples). When the initial investment required for the automation system is considered, the automated method becomes cost-effective after approximately 87 runs with 96 samples each.

## Discussion

This work evaluated the efficiency of automated nucleic acid extraction compared with manual method for processing large sample sets for the detection of *Plasmodium* species by RT-qPCR. In addition, two different RNA preservation methods were evaluated. The results show that the mean Cq values obtained from RT-qPCR targeting the 18S rRNA and DNA of *Plasmodium* spp. in a single reaction were only minimally influenced by RNA preservation solutions (p = 0.686) or extraction methods (p = 0.119). Higher Cq values were observed with the manual method, which might indicate a higher recovery of total nucleic acids with the automated method, however this did not result in a different limit of detection. The flatter slopes observed in the four groups of samples could be a result of sample preparation in which human nucleic acids are not diluted, as the mocked *P. falciparum* dilutions were spiked with human whole blood. This might result in competition of non-target DNA for the extraction and/or primers during PCR, leading to a reduced PCR efficiency. This was not seen when a dilution of extracted DNA was performed, as there the PCR-efficiency was high.

When automated, the magnetic beads-based extraction kit allows the processing of 96 samples simultaneously, thereby increasing throughput, which is especially useful in studies assessing a large number of samples. Once the robot is loaded with samples, reagents and consumables, the time needed to perform the nucleic acid isolation is around one hour, regardless of the number of samples. In contrast, the isolation of nucleic acids using the manual kit takes considerably longer. Manual handling is only faster for small sample sizes of up to seven samples, assuming a trained laboratory person is performing the assay. However, the choice for the robotic system for small-scale sample processing, that do not fulfil a 96-well plate, must consider the potential for plastic waste due to inefficient material utilization. Besides time efficiency and optimization of staff labour, another advantage of the automated extraction is the reduced sample handling, which can avoid contamination between samples when compared to manual extraction with the column-based kit. A drawback is the investment needed to purchase the automation system and plastic consumables, in opposition to column-based kits, that require equipment routinely present in a laboratory setting such as a centrifuge and heating block. It becomes cost-effective after approximately 87 runs, so that the initial investment needed for acquiring the automated system can be a constraint in laboratories with limited resources.

In this study, the automated nucleic acid extraction was tested for detection of *Plasmodium* by RT-qPCR targeting 18S DNA and RNA. Nevertheless, the high-throughput feature of this method makes it feasible to be applied also in other large-scale studies aiming at e.g., surveillance of parasites with mutations associated with antimalarial resistance, assessment of *pfhrp 2/3* deletion status of circulating parasites, evaluation of population genetic diversity and numerous other studies analysing *Plasmodium* molecular markers. When scaled up, these studies have the potential to generate valuable information for malaria control measures and can benefit from automated processes.

The compared RNA preservation solutions did not reveal a difference in the recovered nucleic acids. This makes DNA/RNA Shield a preferred preservation solution, as it does not need to be removed from the sample prior to use of the extraction kit. When extracting nucleic acids from blood samples preserved in RNAlater, an initial step of centrifugation, removal of the supernatant containing RNA later, and dilution with PBS should be carried out so that the extraction can be successfully performed with the column-based kit, as otherwise the columns get clogged.

One limitation of this study is that mock samples derived from *P. falciparum* cultures were utilized, and no clinical samples were tested. PCR inhibitors that could be present in different samples/ sample preparations were therefore not tested and compared. However, future work is planned to implement the automated extraction method for assessing *Plasmodium* prevalence in samples from clinical trials and epidemiological studies.

The QIAamp kit is widely used for nucleic acid isolation from blood samples for detection of *Plasmodium* by PCR [21]. A previous study compared nucleic acid extraction using the QIAamp kit and another automated platform with magnetic particles, the EZ-1 system (Qiagen) and observed concordant results for identification of malaria cases in 48 samples. However, significant lower Cq values were observed with an in-house malaria multiplex qPCR after manual extraction, whereas with a commercial qPCR kit this difference was not observed [22]. Another study compared manual extraction with Chelex and automated extraction with QIAsymphony (Qiagen) from dried blood spots prepared with Dd2 and 3D7 *P. falciparum* cultures. Conversely, although the authors observed comparable qPCR results, samples extracted with the robot presented slightly lower average Cq values in comparison to the manual method. Both methods presented the same sensitivity [23]. In conjunction with these results, the findings of the present study support the use of various automated systems for nucleic acid isolation for detection of *P. falciparum* by qPCR.

## Conclusions

The results presented here demonstrate that automated extraction using magnetic beads offers comparable nucleic acids yields to traditional manual column-based kit when analysed by RT-qPCR targeting *Plasmodium* spp. 18S rRNA and DNA in a single reaction. Therefore, automated extraction of samples preserved in DNA/RNA Shield can be applied with the purpose of optimizing time and staff labour, especially in studies assessing *Plasmodium* infections in a large number of samples.

## Data Availability

The datasets generated during and/or analyzed during the current study are available from the corresponding author on reasonable request.
